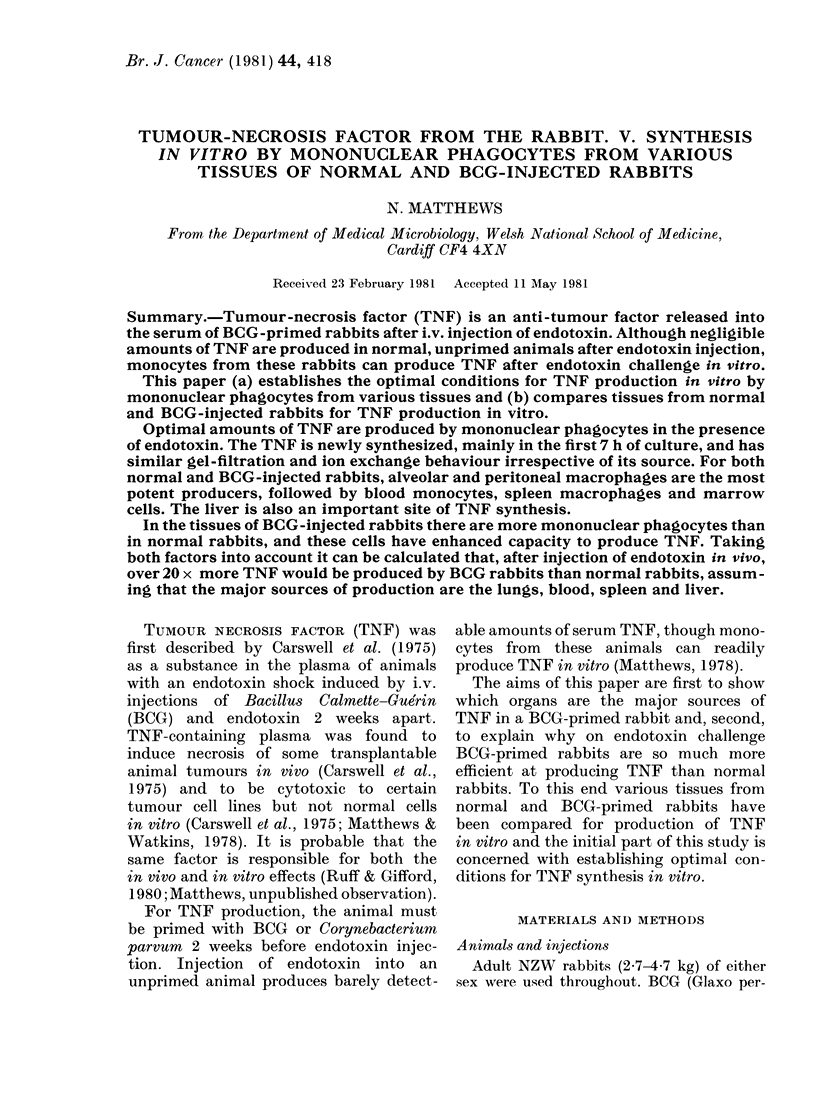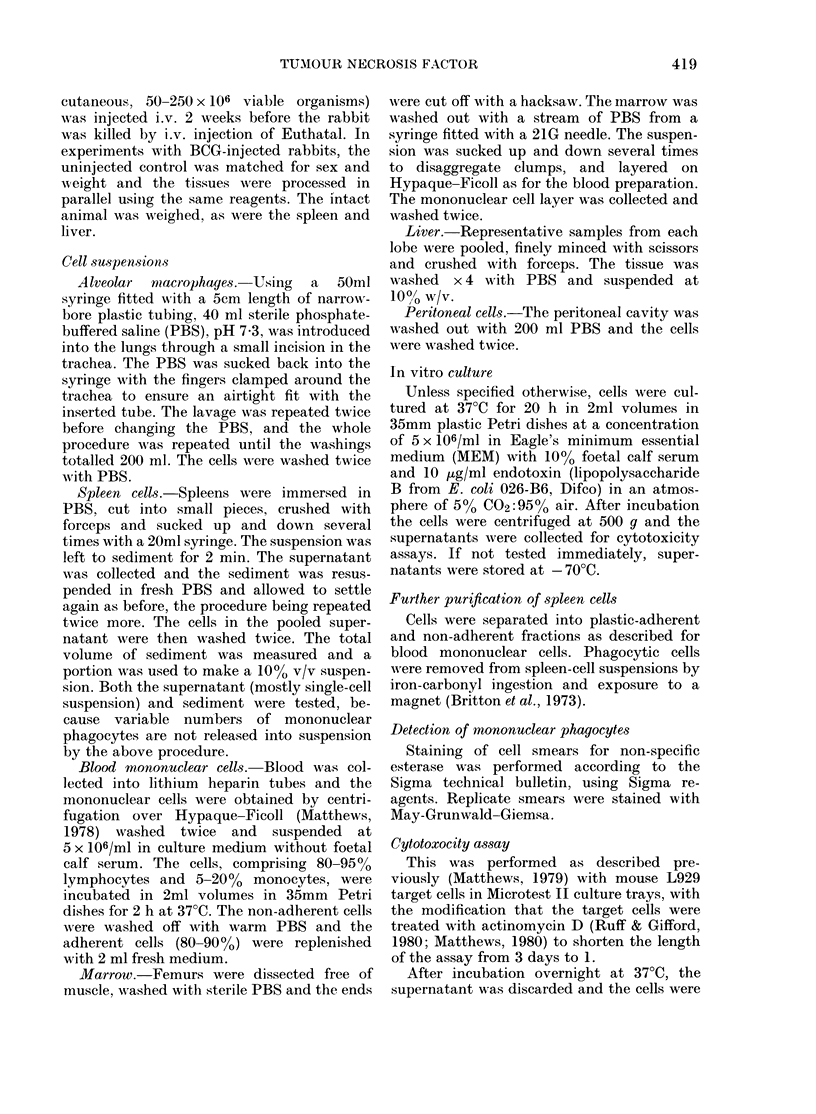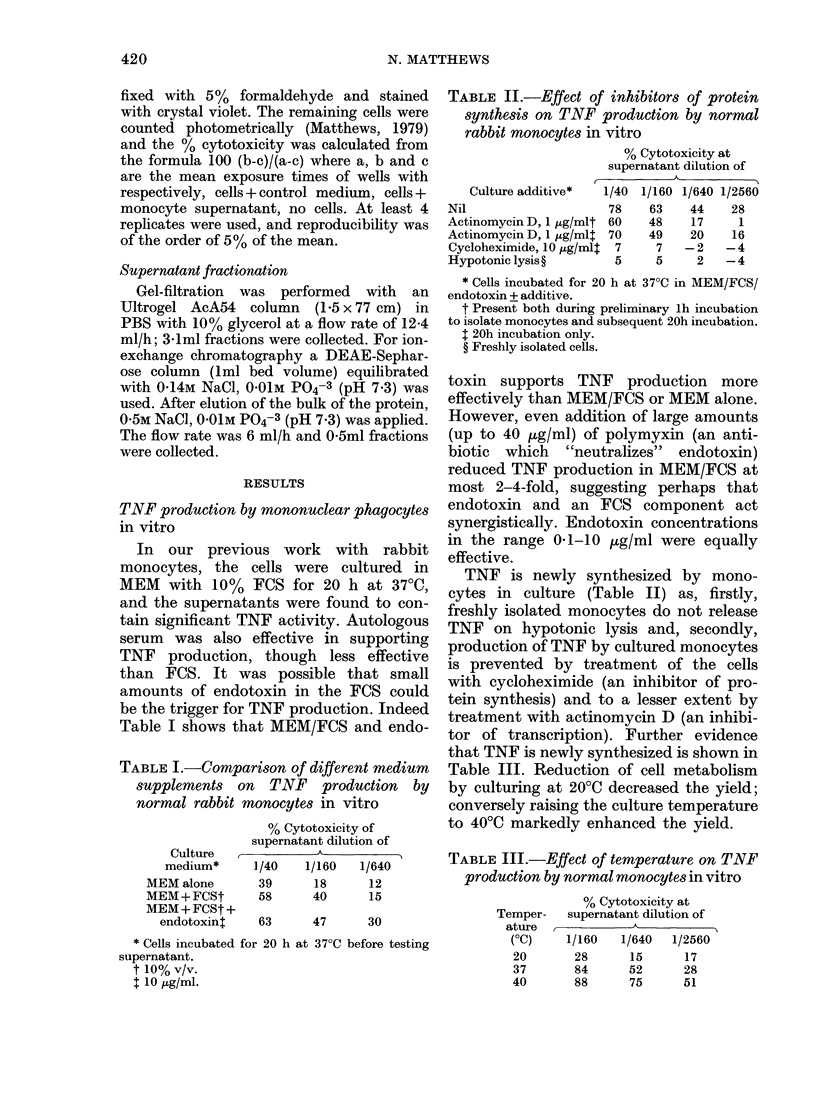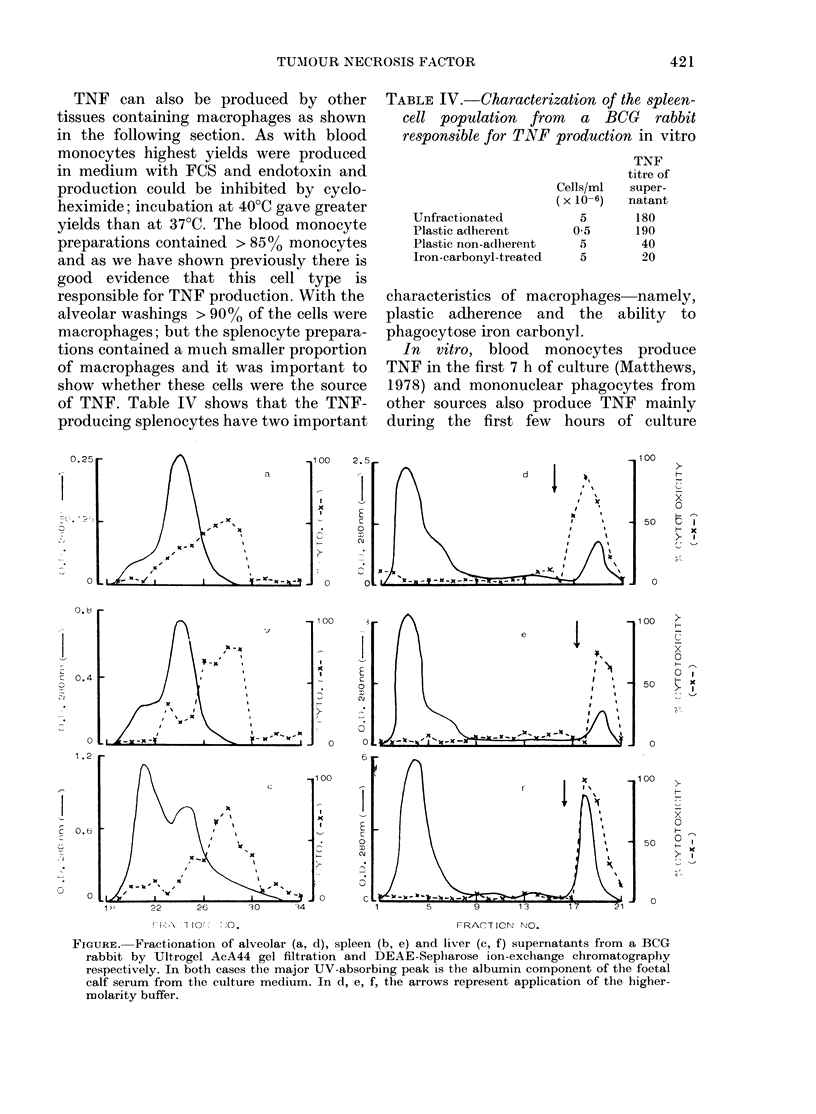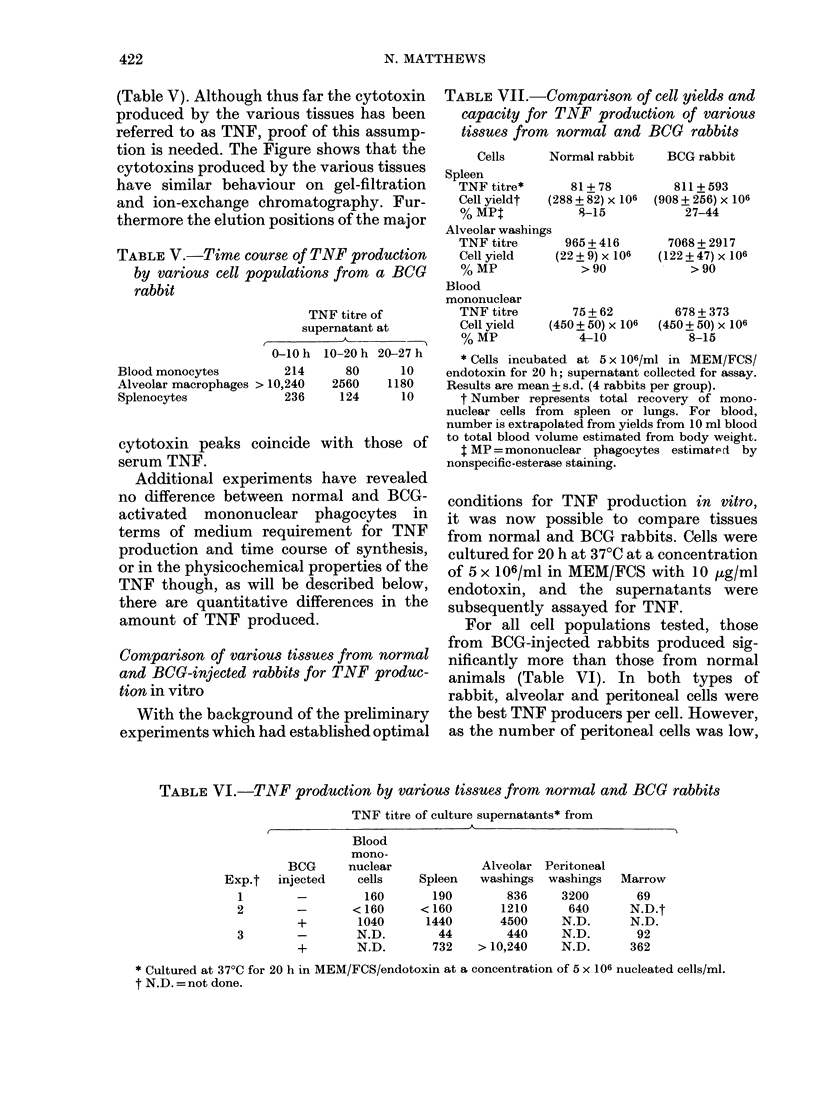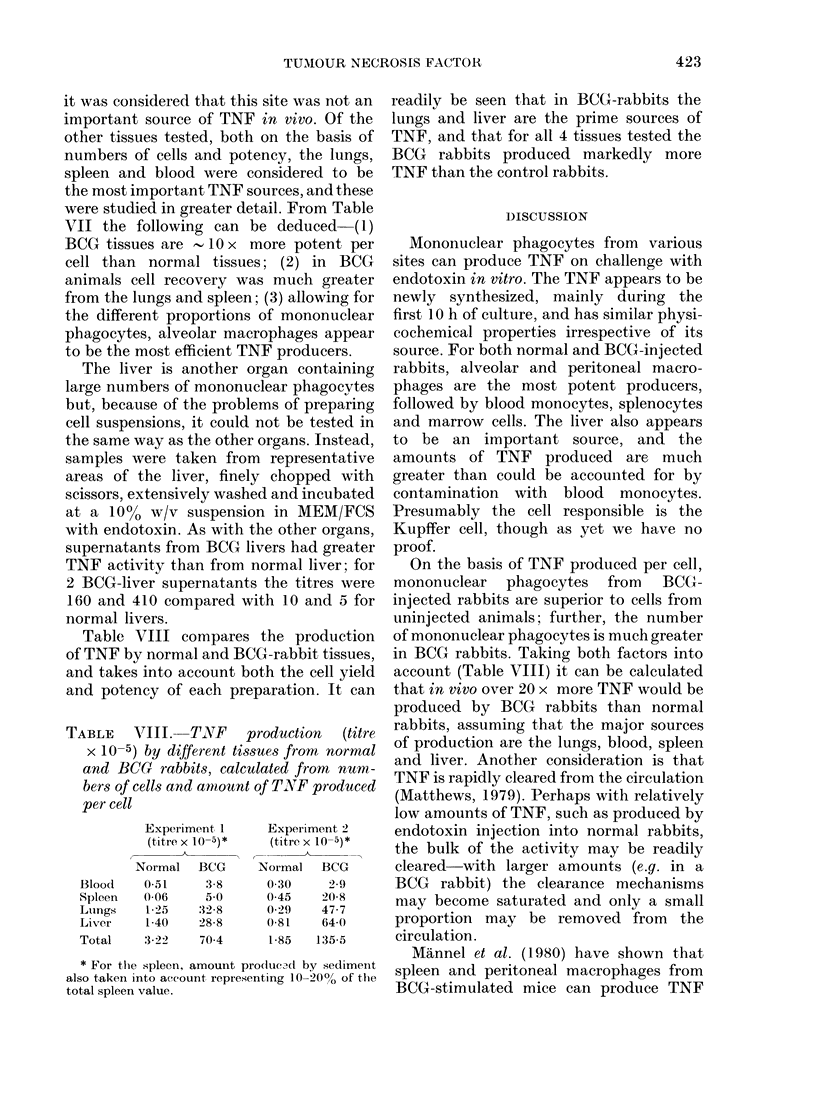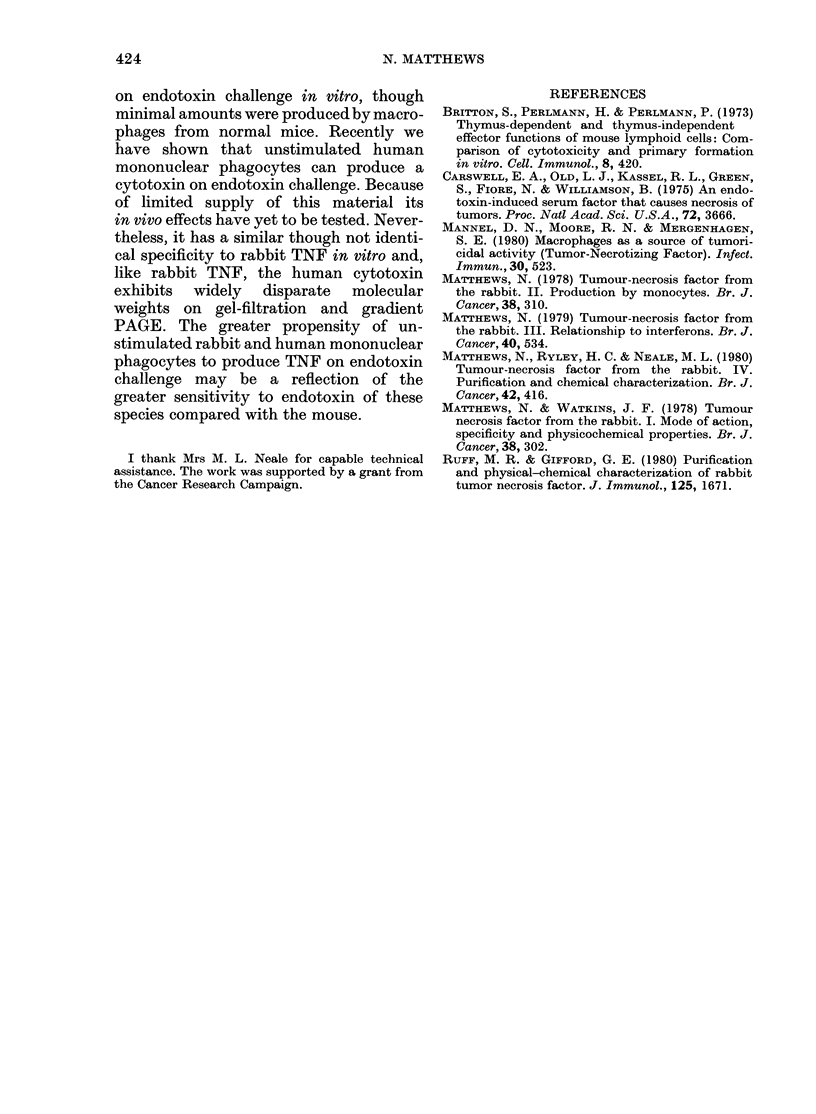# Tumour-necrosis factor from the rabbit. V. Synthesis in vitro by mononuclear phagocytes from various tissues of normal and BCG-injected rabbits.

**DOI:** 10.1038/bjc.1981.200

**Published:** 1981-09

**Authors:** N. Matthews

## Abstract

Tumour-necrosis factor (TNF) is an anti-tumour factor released into the serum of BCG-primed rabbits after i.v. injection of endotoxin. Although negligible amounts of TNF are produced in normal, unprimed animals after endotoxin injection, monocytes from these rabbits can produce TNF after endotoxin challenge in vitro. This paper (a) establishes the optimal conditions for TNF production in vitro by mononuclear phagocytes from various tissues and (b) compares tissues from normal and BCG-injected rabbits for TNF production in vitro. Optimal amounts of TNF are produced by mononuclear phagocytes in the presence of endotoxin. The TNF is newly synthesized, mainly in the first 7 h of culture, and has similar gel-filtration and ion exchange behaviour irrespective of its source. For both normal and BCG-injected rabbits, alveolar and peritoneal macrophages are the most potent producers, followed by blood monocytes, spleen macrophages and marrow cells. The liver is also an important site of TNF synthesis. In the tissues of BCG-injected rabbits there are more mononuclear phagocytes than in normal rabbits, and these cells have enhanced capacity to produce TNF. Taking both factors into account it can be calculated that, after injection of endotoxin in vivo, over 20 X more TNF would be produced by BCG rabbits than normal rabbits, assuming that the major sources of production are the lungs, blood, spleen and liver.


*Br. J. Cancer* (1981) **44**, 418

### TUMOUR-NECROSIS FACTOR FROM THE RABBIT. V. SYNTHESIS *IN VITRO* BY MONONUCLEAR PHAGOCYTES FROM VARIOUS TISSUES OF NORMAL AND BCG-INJECTED RABBITS

N. MATTHEWS

*From the Department of Medical Microbiology, Welsh National School of Medicine, Cardiff CF4 4XN*

Received 23 February 1981 Accepted 11 May 1981

**Summary.—Tumour-necrosis factor (TNF) is an anti-tumour factor released into the serum of BCG-primed rabbits after i.v. injection of endotoxin. Although negligible amounts of TNF are produced in normal, unprimed animals after endotoxin injection, monocytes from these rabbits can produce TNF after endotoxin challenge *in vitro*.**

**This paper (a) establishes the optimal conditions for TNF production *in vitro* by mononuclear phagocytes from various tissues and (b) compares tissues from normal and BCG-injected rabbits for TNF production in vitro.**

**Optimal amounts of TNF are produced by mononuclear phagocytes in the presence of endotoxin. The TNF is newly synthesized, mainly in the first 7 h of culture, and has similar gel-filtration and ion exchange behaviour irrespective of its source. For both normal and BCG-injected rabbits, alveolar and peritoneal macrophages are the most potent producers, followed by blood monocytes, spleen macrophages and marrow cells. The liver is also an important site of TNF synthesis.**

**In the tissues of BCG-injected rabbits there are more mononuclear phagocytes than in normal rabbits, and these cells have enhanced capacity to produce TNF. Taking both factors into account it can be calculated that, after injection of endotoxin *in vivo*, over 20 × more TNF would be produced by BCG rabbits than normal rabbits, assuming that the major sources of production are the lungs, blood, spleen and liver.**

TUMOUR NECROSIS FACTOR (TNF) was first described by Carswell *et al.* (1975) as a substance in the plasma of animals with an endotoxin shock induced by i.v. injections of *Bacillus Calmette–Guérin* (BCG) and endotoxin 2 weeks apart. TNF-containing plasma was found to induce necrosis of some transplantable animal tumours *in vivo* (Carswell *et al.*, 1975) and to be cytotoxic to certain tumour cell lines but not normal cells *in vitro* (Carswell *et al.*, 1975; Matthews & Watkins, 1978). It is probable that the same factor is responsible for both the *in vivo* and *in vitro* effects (Ruff & Gifford, 1980; Matthews, unpublished observation).

For TNF production, the animal must be primed with BCG or *Corynebacterium parvum* 2 weeks before endotoxin injection. Injection of endotoxin into an unprimed animal produces barely detectable amounts of serum TNF, though monocytes from these animals can readily produce TNF *in vitro* (Matthews, 1978).

The aims of this paper are first to show which organs are the major sources of TNF in a BCG-primed rabbit and, second, to explain why on endotoxin challenge BCG-primed rabbits are so much more efficient at producing TNF than normal rabbits. To this end various tissues from normal and BCG-primed rabbits have been compared for production of TNF *in vitro* and the initial part of this study is concerned with establishing optimal conditions for TNF synthesis *in vitro.*

### MATERIALS AND METHODS

### *Animals and injections*

Adult NZW rabbits (2·7–4·7 kg) of either sex were used throughout. BCG (Glaxo percutaneous, 50–250 × $10^6$ viable organisms) was injected i.v. 2 weeks before the rabbit was killed by i.v. injection of Euthatal. In experiments with BCG-injected rabbits, the uninjected control was matched for sex and weight and the tissues were processed in parallel using the same reagents. The intact animal was weighed, as were the spleen and liver.

*Cell suspensions*

*Alveolar macrophages.*—Using a 50ml syringe fitted with a 5cm length of narrow-bore plastic tubing, 40 ml sterile phosphate-buffered saline (PBS), pH 7·3, was introduced into the lungs through a small incision in the trachea. The PBS was sucked back into the syringe with the fingers clamped around the trachea to ensure an airtight fit with the inserted tube. The lavage was repeated twice before changing the PBS, and the whole procedure was repeated until the washings totalled 200 ml. The cells were washed twice with PBS.

*Spleen cells.*—Spleens were immersed in PBS, cut into small pieces, crushed with forceps and sucked up and down several times with a 20ml syringe. The suspension was left to sediment for 2 min. The supernatant was collected and the sediment was resuspended in fresh PBS and allowed to settle again as before, the procedure being repeated twice more. The cells in the pooled supernatant were then washed twice. The total volume of sediment was measured and a portion was used to make a 10% v/v suspension. Both the supernatant (mostly single-cell suspension) and sediment were tested, because variable numbers of mononuclear phagocytes are not released into suspension by the above procedure.

*Blood mononuclear cells.*—Blood was collected into lithium heparin tubes and the mononuclear cells were obtained by centrifugation over Hypaque–Ficoll (Matthews, 1978) washed twice and suspended at 5 × $10^6$/ml in culture medium without foetal calf serum. The cells, comprising 80–95% lymphocytes and 5–20% monocytes, were incubated in 2ml volumes in 35mm Petri dishes for 2 h at 37°C. The non-adherent cells were washed off with warm PBS and the adherent cells (80–90%) were replenished with 2 ml fresh medium.

*Marrow.*—Femurs were dissected free of muscle, washed with sterile PBS and the ends were cut off with a hacksaw. The marrow was washed out with a stream of PBS from a syringe fitted with a 21G needle. The suspension was sucked up and down several times to disaggregate clumps, and layered on Hypaque–Ficoll as for the blood preparation. The mononuclear cell layer was collected and washed twice.

*Liver.*—Representative samples from each lobe were pooled, finely minced with scissors and crushed with forceps. The tissue was washed × 4 with PBS and suspended at 10% w/v.

*Peritoneal cells.*—The peritoneal cavity was washed out with 200 ml PBS and the cells were washed twice.

In vitro *culture*

Unless specified otherwise, cells were cultured at 37°C for 20 h in 2ml volumes in 35mm plastic Petri dishes at a concentration of 5 × $10^6$/ml in Eagle's minimum essential medium (MEM) with 10% foetal calf serum and 10 $\mu$g/ml endotoxin (lipopolysaccharide B from *E. coli* 026-B6, Difco) in an atmosphere of 5% $CO_2$:95% air. After incubation the cells were centrifuged at 500 *g* and the supernatants were collected for cytotoxicity assays. If not tested immediately, supernatants were stored at −70°C.

*Further purification of spleen cells*

Cells were separated into plastic-adherent and non-adherent fractions as described for blood mononuclear cells. Phagocytic cells were removed from spleen-cell suspensions by iron-carbonyl ingestion and exposure to a magnet (Britton *et al.*, 1973).

*Detection of mononuclear phagocytes*

Staining of cell smears for non-specific esterase was performed according to the Sigma technical bulletin, using Sigma reagents. Replicate smears were stained with May-Grunwald–Giemsa.

*Cytotoxocity assay*

This was performed as described previously (Matthews, 1979) with mouse L929 target cells in Microtest II culture trays, with the modification that the target cells were treated with actinomycin D (Ruff & Gifford, 1980; Matthews, 1980) to shorten the length of the assay from 3 days to 1.

After incubation overnight at 37°C, the supernatant was discarded and the cells were fixed with 5% formaldehyde and stained with crystal violet. The remaining cells were counted photometrically (Matthews, 1979) and the % cytotoxicity was calculated from the formula 100 (b-c)/(a-c) where a, b and c are the mean exposure times of wells with respectively, cells + control medium, cells + monocyte supernatant, no cells. At least 4 replicates were used, and reproducibility was of the order of 5% of the mean.

*Supernatant fractionation*

Gel-filtration was performed with an Ultrogel AcA54 column (1·5 × 77 cm) in PBS with 10% glycerol at a flow rate of 12·4 ml/h; 3·1ml fractions were collected. For ion-exchange chromatography a DEAE-Sepharose column (1ml bed volume) equilibrated with 0·14M NaCl, 0·01M $PO_4^{-3}$ (pH 7·3) was used. After elution of the bulk of the protein, 0·5M NaCl, 0·01M $PO_4^{-3}$ (pH 7·3) was applied. The flow rate was 6 ml/h and 0·5ml fractions were collected.

### RESULTS

*TNF production by mononuclear phagocytes* in vitro

In our previous work with rabbit monocytes, the cells were cultured in MEM with 10% FCS for 20 h at 37°C, and the supernatants were found to contain significant TNF activity. Autologous serum was also effective in supporting TNF production, though less effective than FCS. It was possible that small amounts of endotoxin in the FCS could be the trigger for TNF production. Indeed Table I shows that MEM/FCS and endotoxin supports TNF production more effectively than MEM/FCS or MEM alone. However, even addition of large amounts (up to 40 μg/ml) of polymyxin (an antibiotic which "neutralizes" endotoxin) reduced TNF production in MEM/FCS at most 2–4-fold, suggesting perhaps that endotoxin and an FCS component act synergistically. Endotoxin concentrations in the range 0·1–10 μg/ml were equally effective.

TABLE I.—*Comparison of different medium supplements on TNF production by normal rabbit monocytes* in vitro

| Culture medium* | % Cytotoxicity of supernatant dilution of 1/40 | 1/160 | 1/640 |
|---|---|---|---|
| MEM alone | 39 | 18 | 12 |
| MEM + FCS† | 58 | 40 | 15 |
| MEM + FCS† + endotoxin‡ | 63 | 47 | 30 |

* Cells incubated for 20 h at 37°C before testing supernatant.
† 10% v/v.
‡ 10 μg/ml.

TABLE II.—*Effect of inhibitors of protein synthesis on TNF production by normal rabbit monocytes* in vitro

| Culture additive* | % Cytotoxicity at supernatant dilution of 1/40 | 1/160 | 1/640 | 1/2560 |
|---|---|---|---|---|
| Nil | 78 | 63 | 44 | 28 |
| Actinomycin D, 1 μg/ml† | 60 | 48 | 17 | 1 |
| Actinomycin D, 1 μg/ml‡ | 70 | 49 | 20 | 16 |
| Cycloheximide, 10 μg/ml‡ | 7 | 7 | −2 | −4 |
| Hypotonic lysis§ | 5 | 5 | 2 | −4 |

* Cells incubated for 20 h at 37°C in MEM/FCS/endotoxin ± additive.
† Present both during preliminary 1h incubation to isolate monocytes and subsequent 20h incubation.
‡ 20h incubation only.
§ Freshly isolated cells.

TNF is newly synthesized by monocytes in culture (Table II) as, firstly, freshly isolated monocytes do not release TNF on hypotonic lysis and, secondly, production of TNF by cultured monocytes is prevented by treatment of the cells with cycloheximide (an inhibitor of protein synthesis) and to a lesser extent by treatment with actinomycin D (an inhibitor of transcription). Further evidence that TNF is newly synthesized is shown in Table III. Reduction of cell metabolism by culturing at 20°C decreased the yield; conversely raising the culture temperature to 40°C markedly enhanced the yield.

TABLE III.—*Effect of temperature on TNF production by normal monocytes* in vitro

| Temperature (°C) | % Cytotoxicity at supernatant dilution of 1/160 | 1/640 | 1/2560 |
|---|---|---|---|
| 20 | 28 | 15 | 17 |
| 37 | 84 | 52 | 28 |
| 40 | 88 | 75 | 51 |

TNF can also be produced by other tissues containing macrophages as shown in the following section. As with blood monocytes highest yields were produced in medium with FCS and endotoxin and production could be inhibited by cycloheximide; incubation at 40°C gave greater yields than at 37°C. The blood monocyte preparations contained $>85\%$ monocytes and as we have shown previously there is good evidence that this cell type is responsible for TNF production. With the alveolar washings $>90\%$ of the cells were macrophages; but the splenocyte preparations contained a much smaller proportion of macrophages and it was important to show whether these cells were the source of TNF. Table IV shows that the TNF-producing splenocytes have two important characteristics of macrophages—namely, plastic adherence and the ability to phagocytose iron carbonyl.

TABLE IV.—*Characterization of the spleen-cell population from a BCG rabbit responsible for TNF production* in vitro

| | Cells/ml ($\times 10^{-6}$) | TNF titre of supernatant |
|---|---|---|
| Unfractionated | 5 | 180 |
| Plastic adherent | 0·5 | 190 |
| Plastic non-adherent | 5 | 40 |
| Iron-carbonyl-treated | 5 | 20 |

*In vitro,* blood monocytes produce TNF in the first 7 h of culture (Matthews, 1978) and mononuclear phagocytes from other sources also produce TNF mainly during the first few hours of culture

FIGURE.—Fractionation of alveolar (a, d), spleen (b, e) and liver (c, f) supernatants from a BCG rabbit by Ultrogel AcA44 gel filtration and DEAE-Sepharose ion-exchange chromatography respectively. In both cases the major UV-absorbing peak is the albumin component of the foetal calf serum from the culture medium. In d, e, f, the arrows represent application of the higher-molarity buffer.

(Table V). Although thus far the cytotoxin produced by the various tissues has been referred to as TNF, proof of this assumption is needed. The Figure shows that the cytotoxins produced by the various tissues have similar behaviour on gel-filtration and ion-exchange chromatography. Furthermore the elution positions of the major cytotoxin peaks coincide with those of serum TNF.

TABLE V.—*Time course of TNF production by various cell populations from a BCG rabbit*

| | TNF titre of supernatant at | | |
|---|---|---|---|
| | 0–10 h | 10–20 h | 20–27 h |
| Blood monocytes | 214 | 80 | 10 |
| Alveolar macrophages | > 10,240 | 2560 | 1180 |
| Splenocytes | 236 | 124 | 10 |

Additional experiments have revealed no difference between normal and BCG-activated mononuclear phagocytes in terms of medium requirement for TNF production and time course of synthesis, or in the physicochemical properties of the TNF though, as will be described below, there are quantitative differences in the amount of TNF produced.

*Comparison of various tissues from normal and BCG-injected rabbits for TNF production* in vitro

With the background of the preliminary experiments which had established optimal conditions for TNF production *in vitro*, it was now possible to compare tissues from normal and BCG rabbits. Cells were cultured for 20 h at 37°C at a concentration of $5 \times 10^6$/ml in MEM/FCS with 10 $\mu$g/ml endotoxin, and the supernatants were subsequently assayed for TNF.

For all cell populations tested, those from BCG-injected rabbits produced significantly more than those from normal animals (Table VI). In both types of rabbit, alveolar and peritoneal cells were the best TNF producers per cell. However, as the number of peritoneal cells was low,

TABLE VII.—*Comparison of cell yields and capacity for TNF production of various tissues from normal and BCG rabbits*

| Cells | Normal rabbit | BCG rabbit |
|---|---|---|
| Spleen | | |
| TNF titre* | $81 \pm 78$ | $811 \pm 593$ |
| Cell yield† | $(288 \pm 82) \times 10^6$ | $(908 \pm 256) \times 10^6$ |
| % MP‡ | 8–15 | 27–44 |
| Alveolar washings | | |
| TNF titre | $965 \pm 416$ | $7068 \pm 2917$ |
| Cell yield | $(22 \pm 9) \times 10^6$ | $(122 \pm 47) \times 10^6$ |
| % MP | > 90 | > 90 |
| Blood mononuclear | | |
| TNF titre | $75 \pm 62$ | $678 \pm 373$ |
| Cell yield | $(450 \pm 50) \times 10^6$ | $(450 \pm 50) \times 10^6$ |
| % MP | 4–10 | 8–15 |

* Cells incubated at $5 \times 10^6$/ml in MEM/FCS/endotoxin for 20 h; supernatant collected for assay. Results are mean ± s.d. (4 rabbits per group).

† Number represents total recovery of mononuclear cells from spleen or lungs. For blood, number is extrapolated from yields from 10 ml blood to total blood volume estimated from body weight.

‡ MP = mononuclear phagocytes estimated by nonspecific-esterase staining.

TABLE VI.—*TNF production by various tissues from normal and BCG rabbits*

| | | TNF titre of culture supernatants* from | | | | |
|---|---|---|---|---|---|---|
| Exp.† | BCG injected | Blood mononuclear cells | Spleen | Alveolar washings | Peritoneal washings | Marrow |
| 1 | – | 160 | 190 | 836 | 3200 | 69 |
| 2 | – | < 160 | < 160 | 1210 | 640 | N.D.† |
| | + | 1040 | 1440 | 4500 | N.D. | N.D. |
| 3 | – | N.D. | 44 | 440 | N.D. | 92 |
| | + | N.D. | 732 | > 10,240 | N.D. | 362 |

* Cultured at 37°C for 20 h in MEM/FCS/endotoxin at a concentration of $5 \times 10^6$ nucleated cells/ml.

† N.D. = not done.

it was considered that this site was not an important source of TNF *in vivo*. Of the other tissues tested, both on the basis of numbers of cells and potency, the lungs, spleen and blood were considered to be the most important TNF sources, and these were studied in greater detail. From Table VII the following can be deduced—(1) BCG tissues are $\sim 10\times$ more potent per cell than normal tissues; (2) in BCG animals cell recovery was much greater from the lungs and spleen; (3) allowing for the different proportions of mononuclear phagocytes, alveolar macrophages appear to be the most efficient TNF producers.

The liver is another organ containing large numbers of mononuclear phagocytes but, because of the problems of preparing cell suspensions, it could not be tested in the same way as the other organs. Instead, samples were taken from representative areas of the liver, finely chopped with scissors, extensively washed and incubated at a 10% w/v suspension in MEM/FCS with endotoxin. As with the other organs, supernatants from BCG livers had greater TNF activity than from normal liver; for 2 BCG-liver supernatants the titres were 160 and 410 compared with 10 and 5 for normal livers.

Table VIII compares the production of TNF by normal and BCG-rabbit tissues, and takes into account both the cell yield and potency of each preparation. It can readily be seen that in BCG-rabbits the lungs and liver are the prime sources of TNF, and that for all 4 tissues tested the BCG rabbits produced markedly more TNF than the control rabbits.

TABLE VIII.—*TNF production* (*titre* $\times 10^{-5}$) *by different tissues from normal and BCG rabbits, calculated from numbers of cells and amount of TNF produced per cell*

| | Experiment 1 (titre $\times 10^{-5}$)* | | Experiment 2 (titre $\times 10^{-5}$)* | |
|---|---|---|---|---|
| | Normal | BCG | Normal | BCG |
| Blood | 0·51 | 3·8 | 0·30 | 2·9 |
| Spleen | 0·06 | 5·0 | 0·45 | 20·8 |
| Lungs | 1·25 | 32·8 | 0·29 | 47·7 |
| Liver | 1·40 | 28·8 | 0·81 | 64·0 |
| Total | 3·22 | 70·4 | 1·85 | 135·5 |

* For the spleen, amount produced by sediment also taken into account representing 10–20% of the total spleen value.

### DISCUSSION

Mononuclear phagocytes from various sites can produce TNF on challenge with endotoxin *in vitro*. The TNF appears to be newly synthesized, mainly during the first 10 h of culture, and has similar physicochemical properties irrespective of its source. For both normal and BCG-injected rabbits, alveolar and peritoneal macrophages are the most potent producers, followed by blood monocytes, splenocytes and marrow cells. The liver also appears to be an important source, and the amounts of TNF produced are much greater than could be accounted for by contamination with blood monocytes. Presumably the cell responsible is the Kupffer cell, though as yet we have no proof.

On the basis of TNF produced per cell, mononuclear phagocytes from BCG-injected rabbits are superior to cells from uninjected animals; further, the number of mononuclear phagocytes is much greater in BCG rabbits. Taking both factors into account (Table VIII) it can be calculated that *in vivo* over $20\times$ more TNF would be produced by BCG rabbits than normal rabbits, assuming that the major sources of production are the lungs, blood, spleen and liver. Another consideration is that TNF is rapidly cleared from the circulation (Matthews, 1979). Perhaps with relatively low amounts of TNF, such as produced by endotoxin injection into normal rabbits, the bulk of the activity may be readily cleared—with larger amounts (*e.g.* in a BCG rabbit) the clearance mechanisms may become saturated and only a small proportion may be removed from the circulation.

Männel *et al.* (1980) have shown that spleen and peritoneal macrophages from BCG-stimulated mice can produce TNF

on endotoxin challenge *in vitro*, though minimal amounts were produced by macrophages from normal mice. Recently we have shown that unstimulated human mononuclear phagocytes can produce a cytotoxin on endotoxin challenge. Because of limited supply of this material its *in vivo* effects have yet to be tested. Nevertheless, it has a similar though not identical specificity to rabbit TNF *in vitro* and, like rabbit TNF, the human cytotoxin exhibits widely disparate molecular weights on gel-filtration and gradient PAGE. The greater propensity of unstimulated rabbit and human mononuclear phagocytes to produce TNF on endotoxin challenge may be a reflection of the greater sensitivity to endotoxin of these species compared with the mouse.

I thank Mrs M. L. Neale for capable technical assistance. The work was supported by a grant from the Cancer Research Campaign.

### REFERENCES

Britton, S., Perlmann, H. & Perlmann, P. (1973) Thymus-dependent and thymus-independent effector functions of mouse lymphoid cells: Comparison of cytotoxicity and primary formation *in vitro*. *Cell. Immunol.*, **8**, 420.

Carswell, E. A., Old, L. J., Kassel, R. L., Green, S., Fiore, N. & Williamson, B. (1975) An endotoxin-induced serum factor that causes necrosis of tumors. *Proc. Natl Acad. Sci. U.S.A.*, **72**, 3666.

Mannel, D. N., Moore, R. N. & Mergenhagen, S. E. (1980) Macrophages as a source of tumoricidal activity (Tumor-Necrotizing Factor). *Infect. Immun.*, **30**, 523.

Matthews, N. (1978) Tumour-necrosis factor from the rabbit. II. Production by monocytes. *Br. J. Cancer*, **38**, 310.

Matthews, N. (1979) Tumour-necrosis factor from the rabbit. III. Relationship to interferons. *Br. J. Cancer*, **40**, 534.

Matthews, N., Ryley, H. C. & Neale, M. L. (1980) Tumour-necrosis factor from the rabbit. IV. Purification and chemical characterization. *Br. J. Cancer*, **42**, 416.

Matthews, N. & Watkins, J. F. (1978) Tumour necrosis factor from the rabbit. I. Mode of action, specificity and physicochemical properties. *Br. J. Cancer*, **38**, 302.

Ruff, M. R. & Gifford, G. E. (1980) Purification and physical–chemical characterization of rabbit tumor necrosis factor. *J. Immunol.*, **125**, 1671.